# Multiscale Modeling of Fiber Fragmentation Process in Aligned ZnO Nanowires Enhanced Single Fiber Composites

**DOI:** 10.1038/s41598-019-56503-x

**Published:** 2019-12-27

**Authors:** Parisa Marashizadeh, Mohammad Abshirini, Jingyu Wang, Mrinal C. Saha, Yingtao Liu

**Affiliations:** School of Aerospace and Mechanical Engineering, University of Oklahoma, Norman, OK 73019 USA

**Keywords:** Aerospace engineering, Mechanical engineering, Computational methods

## Abstract

A three-dimensional multiscale modeling framework is developed to analyze the failure procedure of radially aligned zinc oxide (ZnO) enhanced single fiber composites (SFC) under tensile loading to understand the interfacial improvement between the fiber and the matrix. The model introduces four levels in the computational domain. The nanoscale analysis calculates the size-dependent material properties of ZnO nanowires. The interaction between ZnO nanowires and the matrix is simulated using a properly designed representative volume element at the microscale. At the mesoscale, the interface between the carbon fiber and the surrounding area is modeled using the cohesive zone approach. A combination of ABAQUS Finite element software and the failure criteria modeled in UMAT user subroutine is implemented to simulate the single fiber fragmentation test (SFFT) at the macroscale. The numerical results indicate that the interfacial shear strength of SFC can be improved up to 99% after growing ZnO nanowires on the fiber. The effect of ZnO nanowires geometries on the interfacial shear strength of the enhanced SFC is also investigated. Experimental ZnO nanowires enhanced SFFTs are performed on the fabricated samples to validate the results of the developed multiscale model. A good agreement between the numerical and the experimental results was observed.

## Introduction

Fiber reinforced polymer composites are increasingly being used in a large number of applications ranging from aerospace systems to automotive, industrial, and consumer products due to their outstanding properties, such as light in weight, high strength to weight ratio, and corrosion protection capability^1,2^. Due to their remarkable mechanical, electrical and thermal properties, nanoparticles, such as multiwall carbon nanotubes (MWCNTs) and zinc oxide (ZnO) nanowires, have been dispersed in the polymer matrix to improve the properties of composites. Aligned ZnO nanowires have been synthesized on carbon fibers^3,4^ and aramid fibers^5^ using the hydrothermal method to enhance the bonding strength between polymers and fibers. Experimental and numerical analysis has been shown that the aligned MWCNTs^6^ and ZnO nanowires^7^ can be employed to enhance the interlaminar and interyarn properties of composites. From the modeling point of view, a representative volume element (RVE) is proposed by Kulkarni *et al*.^8^ for estimating the effective material properties of nanostructured hybrid composites. Kundalwal *et al*.^9,10^ explored the thermal and mechanical properties of the radially aligned CNTs fiber composites at different length scales.

The strength and toughness of composites greatly depend on the properties of the fiber-matrix interface through multiple length scales. Single-fiber composite (SFC) tests, including fiber pull-out test and single fiber fragmentation test (SFFT), have been reported as a useful method to characterize the interfacial properties and damage process at the constituent level^11,12^. Previous research has demonstrated that the microscale fiber breakage in SFFT under tensile load is controlled by interfacial properties, such as interfacial strength and toughness^13,14^.

Two different solid mechanics models have been widely used in the literature to simulate the interfacial properties of composites in SFFTs. The first mechanical model explains the interfacial properties of composites by analyzing the broken fiber lengths in SFFT. For example, the constant shear stress model developed by Kelly and Tyson has been widely used to extract the interfacial properties by calculating the distribution of the fragmented fiber lengths, neglecting the matrix cracks and the debonding around fiber breaks^15^. In the second mechanics model, the interfacial fracture toughness is determined based on the fragmentation process and debonding growth^13^. However, the microscale damage process during the fiber fragmentation process is more complicated than the published models. Therefore, it is necessary to develop new solid mechanics models to better understand the entire damage process for the quantifications of the interfacial properties in SFCs.

SFFTs and related analyses have been reported to study the interfacial properties of MWCNT and ZnO nanowire enhanced composites^3,16,17^. The aligned nanoparticles increased the surface area of the structural fiber, leading to enhanced interfacial properties. However, the current models simplify the nanoparticles enhanced interface as a homogeneous layer between fiber and matrix without considering the size effect, distribution, and orientation of nanoparticles in the matrix. Therefore, it is essential to develop a multiscale modeling approach to fully understand the effect of nanostructures and nanoparticles as the enhancement of interfacial properties in composites.

**Figure 1:**
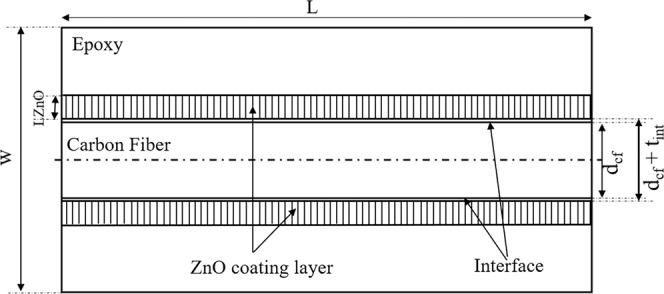
Schematic of enhanced SFC with ZnO coating.

This paper presents a three-dimensional (3-D) multiscale approach to analyze the interfacial properties of aligned ZnO nanowire enhanced SFCs under SFFTs. The schematic of the enhanced single fiber reinforced epoxy model is shown in Fig. 1. Since the studied nanocomposites consist of four different phases in diverse length scales (ZnO nanowires, interface, carbon fiber, and epoxy matrix), the model is developed in multiple length scales described in section 2. Section 3 discusses the fiber fragmentation analysis. Section 4 presents the finite element model used for the analysis of the SFFTs. Detailed simulation results and experimental comparisons are presented in Sections 5 and 6.

## Multiscale Modeling of Enhanced SFCs with Aligned ZnO Nanowires

**Figure 2:**
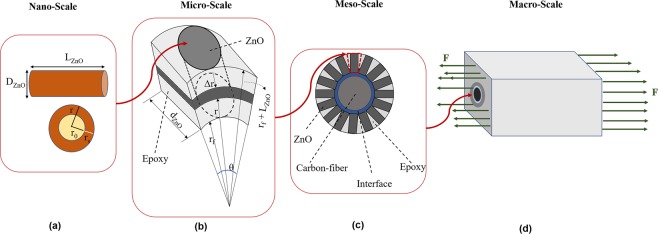
Multiscale modeling of the enhanced SFC (**a**) ZnO nanowire at nanoscale, (**b**) RVE of ZnO reinforced epoxy (microscale), (**c**) interactions of the aligned nanowires on the fiber surface considering the enhanced interface (mesoscale), (**d**) damage analysis of enhanced SFC in the tensile loading (macroscale).

The multiscale analysis and modeling of the enhanced SFCs are described in this section, which is divided into four length scales: (i) single ZnO nanowire at nanoscale, (ii) ZnO reinforced epoxy unit cell at microscale, (iii) interfacial enhancement of aligned ZnO arrays on the carbon fiber at mesoscale, and (iv) the SFFT and fiber fractures at macroscale, as shown in Fig. 2. The modeling results at each length scale are implemented as input for the next length scale.

### Nanoscale analysis of single ZnO nanowire

A core-shell structure is used to model ZnO nanowires and to evaluate the material properties of ZnO nanowires with different geometries. According to the theoretical approach proposed by Chen *et al*.^18^ and verified with experiments, the size-dependent Young’s modulus of ZnO nanowires with diameters in the range of 17–450 nm can be calculated using Eq. 1.

1 $$E={E}_{0}[1+8(\frac{{E}_{s}}{{E}_{0}}-1)(\frac{{r}_{s}}{D}-3\frac{{r}_{s}^{2}}{{D}^{2}}+4\frac{{r}_{s}^{3}}{{D}^{3}}-2\frac{{r}_{s}^{4}}{{D}^{4}})]$$

where *E*_0_ is the modulus of the bulk ZnO material. Considering a core-shell structure of the nanowires as a composite wire, *E*_*s*_ is the surface modulus, *r*_*s*_ is the depth of the shell and *D* is the nanowire diameter. The obtained value of *E*_0_ is 139 GPa, *r*_*s*_ is 4.4 nm, and *E*_*s*_*/E*_0_ is equal to 1.50^18^. Besides, various typical lengths of nanowires in the range of 2.5–14 *µ*m are taken into account in this study, which can control the enhanced ZnO/epoxy coating layer as shown in Fig. 1.

### Microscale modeling of ZnO reinforced epoxy unit cell

In order to study the interfacial enhancement of hybrid SFC, the coating layer (radially aligned ZnO) is modeled as nanowires reinforced epoxy as shown in Fig. 2b,c. A small section of the ZnO/epoxy coating layer with the periodic unit cell can demonstrate the properties of the whole geometry. According to Fig. 2b, a representative volume element (RVE) can be considered in which, a single ZnO nanowire is embedded in the matrix. The maximum ZnO volume fraction in the coating layer can be achieved with the compact radial alignment of the nanowires orthogonal to the carbon fiber as demonstrated in Fig. 2c. Considering an element consists of a single ZnO with the length of (*L*_*ZnO*_) reinforced the epoxy shown in Fig. 2b, the maximum volume fraction of ZnO can be defined as Eq. 2. The detail of this calculation is described in the Appendix.

2 $${v}_{ZnO}=\frac{\pi {d}_{f}}{4({d}_{f}+{L}_{ZnO})}$$

where *d*_*f*_ is the diameter of the carbon fiber and *L*_*ZnO*_ is the length of the ZnO nanowire. The effective material properties of the coating layer can be estimated by the homogenization of this RVE based on the theory of continuum micro-mechanics. The bonding between ZnO and epoxy in this RVE is assumed to be perfect. According to the theory of mechanics, the stress-strain equations for an anisotropic material is defined by Eq. 3.

3 $${\{\varepsilon \}}^{i}={[S]}^{ij}{\{\sigma \}}^{j}\,i,j=1,2\ldots ,6$$

where {*ε*}^*i*^ is the strain vector, {*σ*}^*j*^ is the stress vector, and [*S*]^*ij*^ is the compliance matrix. The compliance matrix can be obtained from Eq. 4. for the orthotropic composite materials^19^.

4 $${[S]}^{ij}=[\begin{array}{cccccc}\frac{1}{{E}_{11}} & -\frac{{\nu }_{21}}{{E}_{22}} & -\frac{{\nu }_{31}}{{E}_{33}} & 0 & 0 & 0\\ -\frac{{\nu }_{12}}{{E}_{11}} & \frac{1}{{E}_{22}} & -\frac{{\nu }_{32}}{{E}_{33}} & 0 & 0 & 0\\ -\frac{{\nu }_{13}}{{E}_{11}} & -\frac{{\nu }_{23}}{{E}_{22}} & \frac{1}{{E}_{33}} & 0 & 0 & 0\\ 0 & 0 & 0 & \frac{1}{{G}_{23}} & 0 & 0\\ 0 & 0 & 0 & 0 & \frac{1}{{G}_{31}} & 0\\ 0 & 0 & 0 & 0 & 0 & \frac{1}{{G}_{12}}\end{array}]$$

where *E*_*ij*_ is Young’s modulus, *G*_*ij*_ is the shear modulus, and *ν*_*ij*_ is the Poisson’s ratio of the orthotropic material in the *ij* = *1, 2, 3* directions, respectively. Assuming 1 is the longitudinal direction, 2 is the in-plane transverse direction, and 3 is the out of plane transverse direction. Considering the volume of the RVE as *V*_*RVE*_, the average strain (${\bar{\varepsilon }}_{ij}$) and stress (${\bar{\sigma }}_{ij}$) of the composite can be calculated from Eqs. 5, 6.

5 $${\bar{\varepsilon }}_{ij}=\frac{1}{{V}_{RVE}}{\int }_{V}{\varepsilon }_{ij}(x,y,z)dV$$

6 $${\bar{\sigma }}_{ij}=\frac{1}{{V}_{RVE}}{\int }_{V}{\sigma }_{ij}(x,y,z)dV$$

In this homogeneous RVE, the total energy of the system (*U*) is obtained from Eq. 7, while the stored energy in the system (U^*^) can be found from Eq. 8.

7 $$U=\frac{1}{2}{\bar{\sigma }}_{ij}{\bar{\varepsilon }}_{ij}{V}_{RVE}$$

8 $${U}^{\ast }=\frac{1}{2}{\int }_{V}{\sigma }_{ij}{\varepsilon }_{ij}dV$$

**Figure 3:**
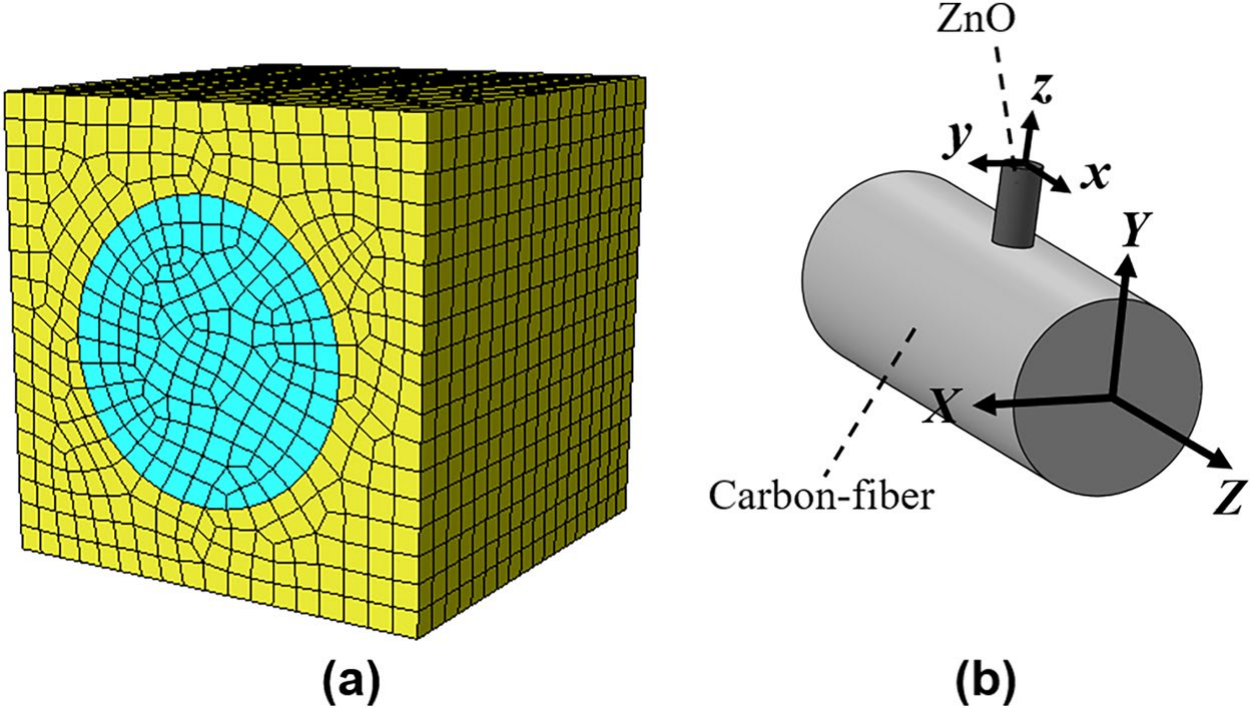
(**a**) FEM model of the RVE for ZnO reinforced epoxy structure, (**b**) relation between the carbon fiber and ZnO coordinate system.

According to the equilibrium state of the strain energy in the system, U^*^− *U* = 0. By applying the different boundary conditions and displacements in the RVE model, and using equilibrium principle of energy, the matrix of elastic compliance can be calculated^20^. More information about extracting the effective material properties of a square cross-section RVE using the continuum mechanics can be found in the ref. ^21^. The approach provided by Omairey, *et al*.^22^ is used in ABAQUS for obtaining the effective material properties of the ZnO nanowires reinforced epoxy. The simulated model of this analysis is shown in Fig. 3a considering the perpendicular orientation of the carbon fiber and ZnO depicted in Fig. 3b, the obtained material properties should be updated based on the coordinate system of the carbon fiber (upper letter) and the ZnO nanowire (lower letter).

### Mesoscale analysis of the fiber/matrix interface

**Figure 4:**
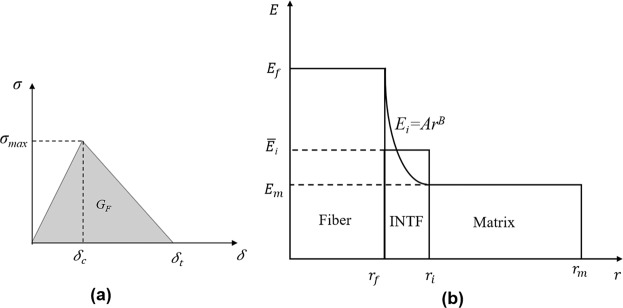
(**a**) The bilinear traction-separation law of the cohesive zone, (**b**) Interface stiffness used in this study.

The adhesion bonding between the carbon fiber and the surrounded area is modeled at mesoscale based on the cohesive zone model and the cohesive elements. In this method, the interface is modeled as a very thin layer. The bilinear traction-separation law is considered to model the stress-displacement of the interface, which has been widely used in the FEM modeling of the carbon fiber reinforced epoxy^23,24^. According to this model, when the stress in the interface layer reaches its strength, the progressive damage initiates and the stress degrades linearly to model the separation of the interface^25^ as shown in Fig. 4a. The maximum stress in the interface is defined as nominal or shear interface strength (*σ*_*max*_) and the fracture energy (*G*_*Ic*_) is described as the area under the bi-linear curve. The stress-displacement relation for different parts of the bilinear traction-separation can be defined by Eq. 9^26^.

9 $$\sigma =(1-D)K\delta $$

where *D* is the damage parameter and *K* is the stiffness in the interface. The *D* parameter is defined with Eq. 10 using the maximum stress criteria for modeling the damage in the interface.

10 $$D=max\{\begin{array}{c}0\\ \frac{(\delta -{\delta }_{c}){\delta }_{t}}{({\delta }_{t}-{\delta }_{c}){\delta }_{t}}\\ 1\end{array}\,\begin{array}{c}\delta \le {\delta }_{c}\\ {\delta }_{c}\le \delta \le {\delta }_{t}\\ \delta \ge {\delta }_{t}\end{array}\}$$

The values of the bilinear parameters can be calculated with the experiments. In the case of carbon fiber and epoxy matrix, 40–60 MPa has been reported for the *σ*_*max*_^24,27^, while the value of *G*_*Ic*_ has been obtained between 100–230 J/m^2 ^^28^. The values of the maximum stress and the fracture energy is considered as (*σ*_*max*_ = 50 MPa, *G*_*Ic*_ = 100 J/m^2^) in this study. Calculation of a specific value for the stiffness with the experimental or analytical analysis is a challenge, and a wide range can be found in the literature. Accordingly, the mathematical approach is implemented here for calculating the interface stiffness based on the material properties of the fiber and the surrounded materials. Different radial variation of the interface modulus across its thickness has been explored in the literature with the boundary condition defined as Eq. 11^29^.

11 $${E}_{i}(r={r}_{f})={E}_{f};{E}_{i}(r={r}_{i})={E}_{m}$$

where *E*_*f*_ and *r*_*i*_ show Young’s modulus and the radius of the fiber, while *E*_*m*_ and *r*_*i*_ describe the modulus of the matrix and the radius of the interface, respectively. The effective stiffness of the interface (${\bar{E}}_{i}$) can be defined as the average of the modulus along with the thickness written in the form of Eq. 12^30,31^. Based on the power-law variation shown in Fig. 4b, the interface modulus is written as *E*_*i*_ = *Ar*^*B*^, and the average interface modulus in this model can be written as Eq. 13^29,30^.

12 $${\bar{E}}_{i}=\frac{1}{{t}_{i}}{\int }_{{r}_{f}}^{{r}_{i}}{E}_{r}(r)dr=\frac{1}{({r}_{i}-{r}_{f})}{\int }_{{r}_{f}}^{{r}_{i}}A{r}^{B}dr$$

13 $$\begin{array}{rcl}{\bar{E}}_{i} & = & \frac{{E}_{f}{r}_{f}}{({r}_{i}-{r}_{f})(B+1)}[{(\frac{{r}_{i}}{{r}_{f}})}^{B+1}-1]\\ A & = & \frac{{E}_{f}}{{{r}_{f}}^{B}};B=\frac{\mathrm{ln}\,{E}_{m}-\,\mathrm{ln}\,{E}_{f}}{\mathrm{ln}\,{r}_{i}-\,\mathrm{ln}\,{r}_{f}}\end{array}$$

### Macroscale analysis of SFFT

**Figure 5:**
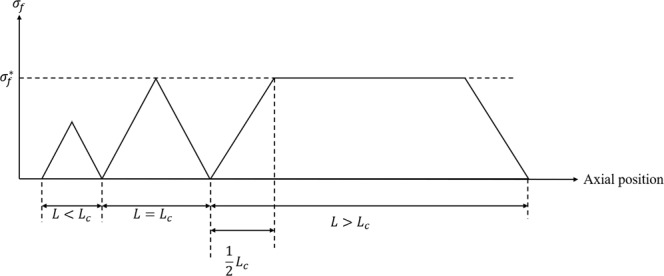
Schematic of the stress distribution in the fiber fragments with different length.

The adhesion bonding between fiber and matrix can be evaluated via the SFFT. The applied tensile load is transferred to the fiber through the interface between matrix and fiber by the shear transferring mechanism. By increasing the load, the fiber breaks into the small fragments after the stress in the fiber exceeds its strength. Each fragment can carry the load to form independent segments and breaks again. This phenomenon continues to form a progressive fiber failure to the point that the segments become too small to break described as saturation level. According to the shear stress model introduced by Kelly-Tyson^32^, the constant interfacial shear strength can be calculated using Eq. 14.

14 $$\tau =\frac{{\sigma }_{f}^{\ast }{r}_{f}}{2({L}_{c}/2)}$$

where *r*_*f*_ and *σ*_f_^*^ are the radius and the ultimate strength of the fiber, respectively. The parameter of (*L*_*c*_/2) is the critical distance from the fiber segment end in which the stress reaches the fiber strength. According to the model provided by Ohsawa *et al*.^33^, the critical length (*L*_*c*_) is defined as a function of the average length of the segments (*L*_*i*_) in the saturation state with *n* fragments as shown in Eq. 15. The mechanism of the stress on different fragments of the fiber is depicted in Fig. 5.

15 $${L}_{c}=\frac{4}{3}(\frac{1}{n}{\sum }_{i=1}^{n}{L}_{i})$$

In order to simulate the SFFT, the failure of the fiber is simulated using the Tsai-Wu failure criterion^34^. The original form of Tsai-Wu expression for orthotropic materials can be written in the form of Eq. 16^35^.

16 $$\begin{array}{rcl}F & = & {F}_{11}{\sigma }_{1}^{2}+{F}_{22}({\sigma }_{2}^{2}+{\sigma }_{3}^{2})+[2{F}_{22}-{F}_{44}]{\sigma }_{2}{\sigma }_{3}+2{F}_{12}{\sigma }_{1}\\  &  & ({\sigma }_{2}+{\sigma }_{3})+{F}_{1}({\sigma }_{1}+{\sigma }_{2})+{F}_{2}{\sigma }_{3}+{F}_{44}{\tau }_{23}^{2}+{F}_{66}({\tau }_{12}^{2}+{\tau }_{13}^{2})\end{array}$$

Assuming the fiber as transversely isotropic material with the tensile strength of *σ*_t_^*^, compressive strength of *σ*_c_^*^, and shear strengths of τ^*^ with the same values in the longitudinal and transverse direction, the *F*_*ij*_ coefficient can be written as Eq. 17.

17 $${F}_{11}={F}_{22}=\frac{1}{{\sigma }_{t}^{\ast }{\sigma }_{c}^{\ast }},{F}_{1}={F}_{2}=(\frac{1}{{\sigma }_{t}^{\ast }}-\frac{1}{{\sigma }_{c}^{\ast }}),{F}_{44}={F}_{66}=\frac{1}{{({\tau }^{\ast })}^{2}},{F}_{12}=0$$

**Figure 6:**
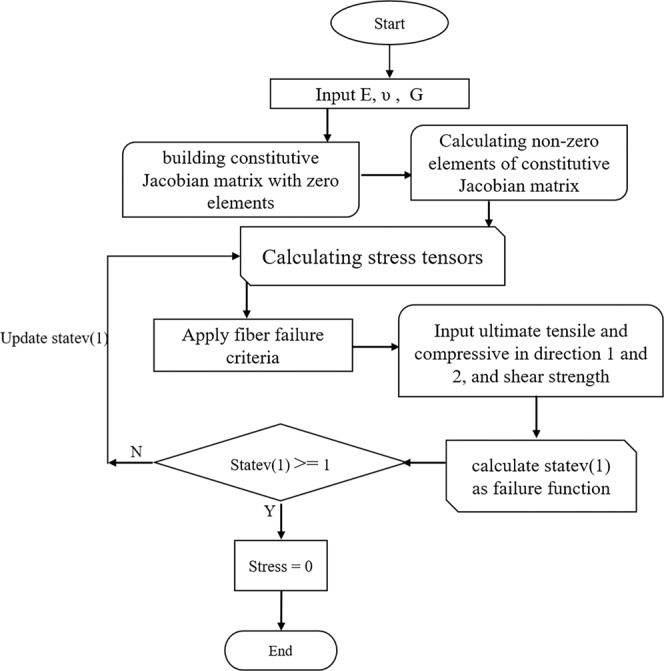
Flowchart of the UMAT subroutine used for the damage analysis.

The magnitude of *F* is called as a reference to the failure situation in which, the material is safe when *F* has a magnitude of smaller than 1, and the failure occurs if *F* equals to 1. The *F* factor is defined and tracked using UMAT user subroutine programmed in FORTRAN. When the damage occurs, the related element cannot carry the load while other elements in the model are active, which breaks the fiber in different segments. The general steps of the damage analysis are depicted in Fig. 6.

## FEM Analysis of SFFT

**Figure 7:**
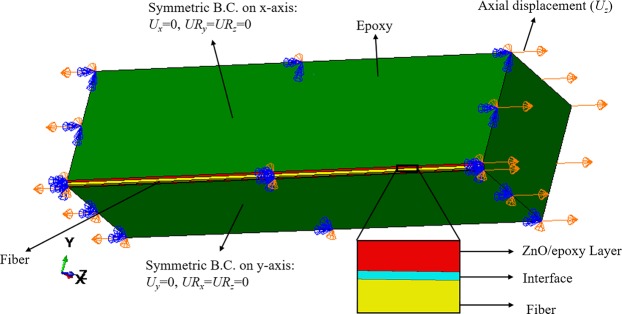
Simulated SFC in FEM with related boundary conditions.

A 3-D model of single carbon fiber embedded in the epoxy is simulated. In order to minimize the computational cost, a quarter of a 3-D composite beam is modeled considering two symmetric planes. Based on the symmetry on the x-plane, the displacement in the x-axis (*U*_*x*_), rotation around y-axis (*UR*_*y*_), and rotation around the z-axis (*Ur*_*z*_) are set to zero. The displacement in the y-axis (*U*_*y*_), rotation around the x-axis (*UR*_*x*_), and rotation around the z-axis (*Ur*_*z*_) are zero for the y-symmetric plane. The axial displacement load is applied to the x-faces as shown in Fig. 7. A thin layer of interface is considered between the fiber and matrix based on the cohesive zone concept to model the adhesive bonding. The FEM analysis of the SFC performed in the ABAQUS package consists of two stages, including bare fiber and enhanced fiber. In the bare SFC, carbon fiber with 7 μm diameter is placed in the center of a cubic matrix with a width of 0.14 mm and a length of 0.5 mm. It has been shown that the interface thickness has a negligible effect on the utilization of the cohesive elements while a value close to zero is suggested^36,37^. Hence, an interface thickness of 0.01 μm is connected to the fiber and epoxy using “tie constraint”. In the enhanced fiber model, the carbon fiber with the same geometry is coated with a layer of ZnO/epoxy composite embedded in the same geometry of matrix to form the four geometry phases. The ZnO nanowires are aligned radially on the surface of the fiber. Accordingly, the thickness of this coating layer is controlled by the length of the ZnO. The material properties of the carbon fiber and epoxy used in this analysis are described in Table 1^38^. The tensile, compressive, and shear strength in this table are shown with *σ*_t_^*^, *σ*_c_^*^, and τ^*^, respectively. The simulated SFC with the defined boundary conditions is depicted in Fig. 7. An 8-node linear brick element with improved surface stress visualization (C3D8S) is utilized for the matrix, ZnO/epoxy layer, and the fiber. An 8-node 3-D cohesive element (COH3D8) is used to model the interface.

**Table 1:** Material properties of the carbon fiber and the epoxy matrix.

Material	*E*_11_ (GPa)	*E*_22_ (GPa)	*ν*_12_	*ν*_23_	*G*_12_ (GPa)	*G*_23_ (GPa)	*σ*_t_^*^ (MPa)	*σ*_c_^*^ (MPa)	τ^*^ (MPa)
Carbon fiber	232	14	0.2	0.25	0.28	5.6	2000	1450	1200
Epoxy	3.5	—	0.3	—	—	—	—	—	—

## Multiscale Modeling Results of SFFT

### Homogenization of the ZnO/epoxy coating layer

The appropriate RVE is considered to calculate the effective material properties of the ZnO/epoxy composite layer. The maximum volume fraction of 57.87% is calculated from Eq. 2 for the ZnO with a length of 2.5 μm. The cubic RVE based on this volume fraction is simulated in ABAQUS (Fig. 3a) considering different diameters and the FEM homogenization is performed. The modulus of the related nanowire diameters extracted from Eq. 1 is implemented in the homogenization analysis. The results for nanowires with diameters of 17, 75, 150, 450 nm are depicted in Table 2. Besides, assuming a constant diameter of 17 nm, the effective material properties of four different nanowire lengths (L =  2.5, 5, 10, 14 μm) were calculated as shown in Table 3. Due to the radial arrangement of nanowires on the fiber, the volume fraction of ZnO in the ZnO/epoxy layer is reducing by increasing the length of the nanowires according to Eq. 2. Based on the relation between the ZnO and the carbon fiber longitudinal axis described in Fig. 3b, the appropriate material properties calculated from the RVE homogenization is used to simulate the ZnO/epoxy coating layer in the enhanced SFC.

**Table 2:** Effective material properties for different ZnO diameter.

*D*_ZnO_ (nm)	*E*_11_ (GPa)	*E*_22_ (GPa)	*E*_33_ (GPa)	*ν*_21_	*ν*_23_	*ν*_31_	*G*_12_ (GPa)	*G*_13_ (GPa)	*G*_23_ (GPa)
17	7.075	7.075	68.120	0.343	0.027	0.260	2.117	2.573	2.573
75	7.023	7.022	55.881	0.342	0.035	0.276	2.111	2.558	2.558
150	7.002	7.003	52.022	0.341	0.038	0.280	2.109	2.552	2.553
450	6.973	6.973	48.728	0.340	0.041	0.288	2.105	2.543	2.543

**Table 3:** Effective material properties for different ZnO length.

*L*_ZnO_ (µm)	*ν*_ZnO_ (%)	*E*_11_ (GPa)	*E*_22_ (GPa)	*E*_33_ (GPa)	*ν*_21_	*ν*_23_	*ν*_31_	*G*_12_ (GPa)	*G*_13_ (GPa)	*G*_23_ (GPa)
2.5	57.8	14.833	14.850	119.025	0.215	0.233	0.233	3.443	5.009	5.009
5	45.8	11.394	11.395	103.374	0.260	0.242	0.242	2.865	3.944	3.944
10	32.3	7.075	7.075	68.120	0.343	0.260	0.260	2.117	2.573	2.573
14	26.2	6.145	6.145	55.868	0.365	0.267	0.267	1.939	2.259	2.259

### Stress distribution along the fiber

**Figure 8:**
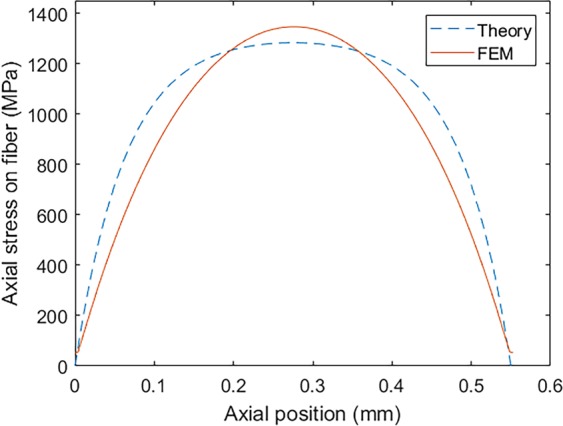
Stress distribution on the bare fiber calculated from FEM and theory.

Considering the material properties and the cohesive zone model described in the previous sections, the stress transferred into the fiber from the matrix is calculated in different situations. In order to evaluate the mechanism of the load transferring, the stress distribution along the fiber length is evaluated for the case of bare SFC. This trend can be compared with the shear-lag theory proposed by Cox^39^. According to this approach, the stress distribution on the fiber can be extracted using Eqs. 18, 19.

18 $${\sigma }_{f}={E}_{f}{\varepsilon }_{m}[1-\frac{\cosh (\frac{nz}{{r}_{f}})}{\cosh (\frac{nL}{2{r}_{f}})}]$$

19 $$n=\sqrt{\frac{2{E}_{m}}{{E}_{f}(1+{\upsilon }_{m})\mathrm{ln}(\frac{1}{{V}_{f}})}}$$

where *r*_*f*_ is the radius of the fiber and *ε*_*m*_ is the applied strain and *V*_*f*_ is the volume fraction of the fiber. Besides, *E*_*f*_ and *E*_*m*_ are Young’s modulus of the fiber and the matrix, respectively. The results calculated by the theory and the FEM for the 0.5% applied strain is shown in Fig. 8. It can be observed that the stress reaches its maximum value at the center of the fiber and degrades to the zero value at the two ends. Besides, the maximum value of the stress in the fiber calculated by FEM is fairly correlated with the theory.

### Fiber fragmentation results

The mesh dependency analysis was first performed to find the proper mesh size for the FEM of SFFT. Hence, the maximum axial stress on the fiber under 0.6% applied strain is obtained for different mesh density as shown in Table 4. Since the relative difference in the FEM results for the 18836 total element numbers is 0.6%, this mesh density is chosen for the analysis.

**Table 4:** Mesh density effects on the fiber’s maximum stress.

**Total number of elements**	756	1728	2506	18836	30326	62380
Max axial stress on the fiber (MPa)	900	1350	1611	1678	1669	1667

**Figure 9:**
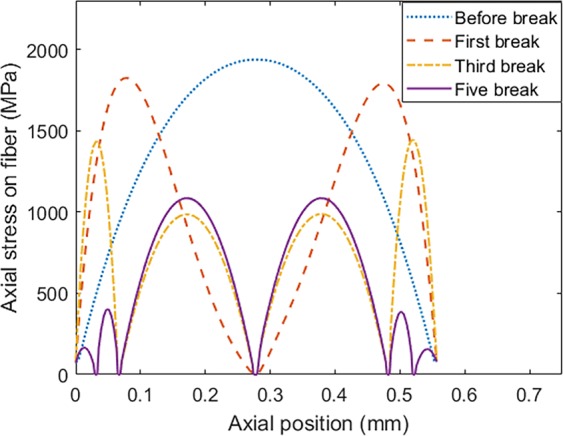
Stress re-distribution along the fiber axis after different fragmentations.

The maximum axial stress on the fiber is increased by improving the applied displacement load on the composite and the failure occurs when the damage model described in the previous section is satisfied. After the failure, the fiber breaks into two different segments each of which can carry the load separately up to the critical state. The stress distribution along the fiber axis at different state of fracture, including before the first break, after the first break, after the third break, and after the five breaks is calculated for the enhanced SFC with *d*_*ZnO*_ = 17 nm and *L*_*ZnO*_ = 2.5 μm as shown in Fig. 9. It can be observed that the fracture occurs in the middle of each segment, while the stress distribution in the smaller segments shows similar trend along the fiber axis.

**Figure 10:**
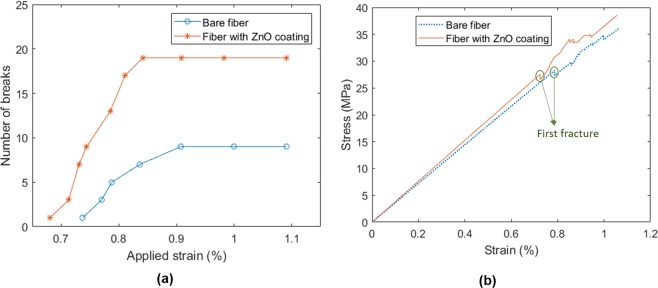
(**a**) Fiber fragmentation density and (**b**) stress-strain curve for the bare and enhanced SFC.

The constitutive failure is continuing up to the state that the segment length in the fiber is small for the stress to reaches the fiber strength. In order to explore the effect of the enhanced interface to the load-carrying properties of the fiber, the density of the fiber fragmentation in the bare fiber is compared to the results of the fiber with the ZnO (*d* = 17 nm and *L* = 2.5 μm) coating layer as illustrated in Fig. 10a. According to this figure, the number of fractures in the enhanced fiber (n = 19) is almost doubled of the bare fiber (n = 9), which means more load is transferred to the fiber through the enhanced interface. Besides, according to this improvement, the first fiber fracture occurred at the lower applied strain in the case of enhanced fiber compared with the bare one. The total number of breaks at the saturated state is used for calculation of the interfacial strength from Eqs. 14, 15. Accordingly, the interfacial shear strength for the bare carbon fiber is 95.5 MPa compared to 190 MPa for the enhanced fiber. In other words, by adding the ZnO/epoxy composite layer on the surface of the carbon fiber, the interfacial shear strength is enhanced by 99%. In addition, the stress-strain curve for the SFC sample is shown in Fig. 10b for the bare and the enhanced fiber. The degradation in the slope of the stress-strain curve indicates the fracture in the fiber. The area where the first fracture happened is marked as an example for both samples. Besides, according to the stronger interface, the first fracture occurs at lower strain compared to the bare sample.

### Effect of the ZnO nanowire geometries

**Figure 11:**
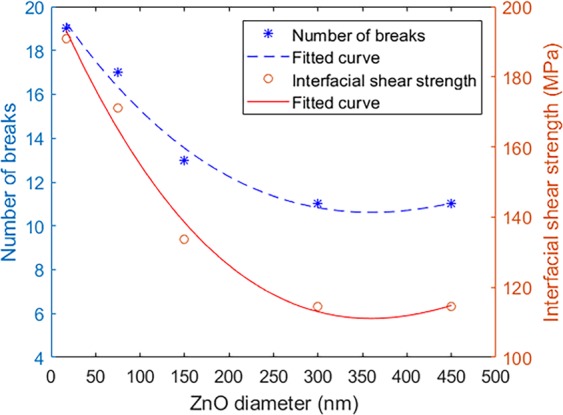
The density of the fiber fragmentation and the interfacial shear strength for different nanowires diameter.

The effect of ZnO nanowires geometry on the enhancement of the interface between fiber and matrix was evaluated. In this regard, the effective material properties for the nanowire with various diameter in the range of 17–450 nm extracted from the homogenization section was implemented in this analysis. The density of the fiber fragmentation calculated from FEM for the different diameter is illustrated in Fig. 11. The interfacial shear strength for each diameter was calculated as shown in this figure. It can be seen that the thin ZnO nanowires result in an increased number of fragmentations (stronger interface). The nonlinear trend indicates the large impact on the interface enhancement when thinner nanowire is applied. The interfacial shear strength for the 17 nm diameter is 190 MPa compared with the 114 MPa for the nanowires with 300 nm diameter. In other words, the interface between fiber and epoxy is stronger when smaller ZnO is used.

**Figure 12:**
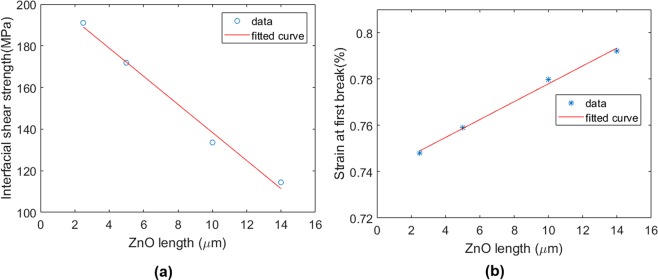
Length effect of the nanowires on (**a**) interfacial shear strength, (**b**) strain at the first fracture.

In addition, the length effect of the nanowires on the interface was explored. The effective material properties of the coating layer with ZnO length of 2.5, 4, 10, and 14 μm is implemented for FEM based on Table 3. By extracting the number of fragmentations, the interfacial shear strength is calculated for each geometry as it is shown in Fig. 12a. It can be observed that the interface is weakened almost linearly by increasing the length of the nanowire. This behavior can be also observed in Fig. 12b in which the strain at the first fracture is shown for different lengths. From this figure, the first fracture occurred earlier in the shorter nanowires based on the stronger interface that leads to transferring more load from the matrix to the fiber.

## Experiments

**Figure 13:**
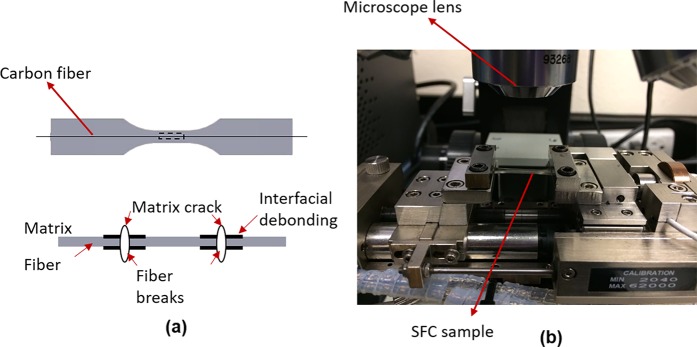
(**a**) Schematic of the sample and possible damages in the SFFT, (**b**) SFFT experimental setup.

SFFT experiments were carried out in this study to validate the interfacial properties of SFC using either bare carbon fiber or ZnO nanowires enhanced carbon in SFC. Atomic layer deposition (ALD) and hydrothermal methods were employed to synthesize the aligned ZnO nanoarrays on carbon fiber fabrics. The detailed nanowire synthesis procedure has been reported in the author’s recent publication^40^. The ZnO nanowires have an average length of 0.98 µm and an average diameter of 24 nm. SFC specimens were fabricated by suspending a fiber along the axis of a silicone dogbone mold. The gauge length of the samples is 15 mm, width is 1.91 mm and the average thickness is about 1.02 mm. The schematic of the single-fiber fragmentation sample and the failure in the SFC are shown in Fig. 13a. The mold cavities were then filled with an epoxy resin (EPON 862/Curing Agent 9553: 100/17.6 by weight) and cured at the elevated temperature for 6 hours at 120 °C. The specimens were tested in a screw-driven micro-tensile machine (Fig. 13b) under a constant displacement rate of 0.2 mm/min, and the fragmentation process was recorded using an optical microscope and polarized light. The specimen was unloaded after the saturation point was reached, and the number of fragments was measured by taking pictures under polarized light.

**Figure 14:**
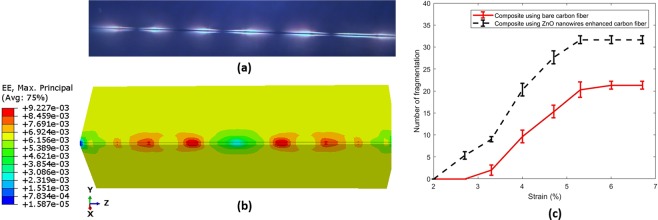
(**a**) Typical micrograph of SFFT showing the fiber brake and damages around the crack tip, (**b**) redistribution of strain around the fracture area obtained from FEM, (**c**) experimental fragmentations density for bare and ZnO coating fiber.

Figure 14(a) shows a typical optical micrograph after tensile testing taken under polarized light. The experiments show that growing the ZnO nanowires has a negligible effect on the fiber’s tensile strength. Fiber breaks, matrix cracks, and interfacial debonding can be observed from the micrograph. The epoxy matrix is optically isotropic but becomes anisotropic due to local stress concentration. In addition, the interfacial debonding between fiber and matrix are highlighted in bright light due to the optically anisotropic matrix in the debonding area. In the FEM, the strain redistribution around the fiber fracture area results in stress/strain concentration on the matrix. These critical areas obtained from the nine fragmentations of the FEM model can be compared with the damage zone of the fiber/matrix in the microscope image as shown in Fig. 14a,b. The experimental results of the number of fractures with the applied strain are shown in Fig. 14c. It can be observed that the average number of fragmentations are increased in the ZnO nanowires enhanced SFC. This result demonstrates that growing ZnO nanowires on the fiber results in a strong interface enhancing the bonding between the carbon fiber and the epoxy matrix. The stronger interface leads to the more efficient load transferring from the matrix to the fiber in the SFFT for the enhanced fiber. Hence, the surface area of each fiber segment has enough shear load to transfer to the fiber to exceeds the strength and causes more fractures.

The average number of fragmentations is 33 and 20 for the hybrid and bare fiber, respectively. The experiments show 88% increase in the interfacial shear strength of the enhanced fiber compared to the bare fiber assuming the same fiber tensile strength, and growth of 0.98 µm in the diameter of the enhanced fiber. The numerical analysis of the radially aligned ZnO nanowires with the similar length and diameter indicates 99% growth of the interfacial shear strength. Considering the possible source of errors in this level of testing, it can be claimed that there is an acceptable agreement between the experimental and the numerical results.

## Conclusion

Multiscale damage analysis of the enhanced single fiber composite is investigated in this study. The ZnO nanowires with various geometries explored at the nanoscale are modeled as radially aligned nanowires on the carbon fiber for improving the interfacial bonding between fiber and matrix. Homogenization analysis of the proposed RVE with various geometries is implemented at the microscale to evaluate the effective material properties of the ZnO/epoxy coating layer. At mesoscale, the maximum volume fraction of the aligned ZnO on the fiber surface is explored. The Cohesive elements with bi-linear traction-separation behavior are used to simulate the interfacial bonding between the enhanced fiber and the matrix. The damage analysis of single enhanced fiber composite is performed using UMAT user subroutine at the macroscale. By comparing the results of the bare fiber with the enhanced fiber, it is concluded that the interfacial shear strength can be improved by 99% with growing ZnO on the fiber. The length and diameter effect of ZnO with common range on the interfacial strength is investigated. It is observed that smaller diameter can result in stronger interfacial bonding between fiber and matrix, which transfers the load from matrix to fiber more efficiently. Considering the effect of nanowire’s length on the ZnO volume fraction in the coating layer, it is shown that shorter nanowires can result in a stronger interface. Experimental results validated the interfacial enhancement using the aligned ZnO nanowires in SFC.

## Appendix

Figure 2b shows an RVE consisting of one ZnO grown on fiber and embedded in the matrix. The maximum possible volume fraction of ZnO in the coating layer can be achieved by the compact orientation of nanowires in which, the thickness of RVE is equal to the diameter of ZnO, its width is the average distance between two nanowires, and its height is the length of ZnO (Fig. 2). The volume fraction of ZnO at any location of the RVE in the coating layer can be written by Eq. A1.

A1 $${\bar{v}}_{ZnO}=\frac{\pi {(\frac{{d}_{ZnO}}{2})}^{2}\Delta r}{\pi [{(r+\Delta r)}^{2}-{r}^{2}]{d}_{ZnO}(\frac{1}{n})}\approx \frac{n(\Delta r){d}_{ZnO}^{2}}{8r\Delta r{d}_{ZnO}}=\frac{n{d}_{ZnO}}{8r}$$

Considering *n* as the number of ZnO grown on the fiber, the homogenized ZnO volume fraction in the RVE is obtained from Eq. A2.

A2 $$\begin{array}{c}{\upsilon }_{ZnO}=\frac{1}{{V}_{RVE}}\mathop{\int }\limits_{V}{\bar{v}}_{ZnO}dV=\frac{1}{\pi [{({r}_{f}+{L}_{ZnO})}^{2}-{{r}_{f}}^{2}]{d}_{ZnO}(\frac{1}{n})}{\int }_{{r}_{f}}^{{r}_{f}+{L}_{ZnO}}\frac{n{d}_{ZnO}}{8r}r\theta {d}_{ZnO}dr=\frac{{n}^{2}\theta {d}_{ZnO}}{8\pi (2{r}_{f}+{L}_{ZnO})}\end{array}$$

It is obvious that *nθ* = 2*π, n* = *πd*_*f*_/*d*_*ZnO*_. Hence, the ZnO maximum volume fraction can be re-written as Eq. A3.

A3 $${v}_{ZnO}=\frac{\pi {d}_{f}}{4({d}_{f}+{L}_{ZnO})}$$
